# Selection Signatures in Four Lignin Genes from Switchgrass Populations Divergently Selected for *In Vitro* Dry Matter Digestibility

**DOI:** 10.1371/journal.pone.0167005

**Published:** 2016-11-28

**Authors:** Shiyu Chen, Shawn M. Kaeppler, Kenneth P. Vogel, Michael D. Casler

**Affiliations:** 1 Department of Agronomy, University of Wisconsin-Madison, Madison, Wisconsin, United States of America; 2 Department of Energy, Great Lakes Bioenergy Research Center, Madison, Wisconsin, United States of America; 3 USDA-ARS, Grain, Forage, and Bioenergy Research Unit, Lincoln, Nebraska, United States of America; 4 Department of Agronomy & Horticulture, University of Nebraska, Lincoln, Nebraska, United States of America; 5 USDA-ARS, U.S. Dairy Forage Research Center, Madison, Wisconsin, United States of America; Iowa State University, UNITED STATES

## Abstract

Switchgrass is undergoing development as a dedicated cellulosic bioenergy crop. Fermentation of lignocellulosic biomass to ethanol in a bioenergy system or to volatile fatty acids in a livestock production system is strongly and negatively influenced by lignification of cell walls. This study detects specific loci that exhibit selection signatures across switchgrass breeding populations that differ in *in vitro* dry matter digestibility (IVDMD), ethanol yield, and lignin concentration. Allele frequency changes in candidate genes were used to detect loci under selection. Out of the 183 polymorphisms identified in the four candidate genes, twenty-five loci in the intron regions and four loci in coding regions were found to display a selection signature. All loci in the coding regions are synonymous substitutions. Selection in both directions were observed on polymorphisms that appeared to be under selection. Genetic diversity and linkage disequilibrium within the candidate genes were low. The recurrent divergent selection caused excessive moderate allele frequencies in the cycle 3 reduced lignin population as compared to the base population. This study provides valuable insight on genetic changes occurring in short-term selection in the polyploid populations, and discovered potential markers for breeding switchgrass with improved biomass quality.

## Introduction

Over the last decade, biomass energy consumption has increased more than 60%, driven by biofuel production, mainly in the form of bioethanol [1]. Switchgrass-based ethanol production contributes to energy diversification and environmental sustainability [2]. Ethanol production from switchgrass biomass produces 540% more renewable energy than nonrenewable energy consumed during the production process, while reducing greenhouse-gas emissions by 94% compared to gasoline [3]. However, due to the hydrophobicity of lignin and the cross-linking between lignin and hemicellulose in the cell walls, pretreatments are required to facilitate the enzymatic hydrolysis of cellulose and hemicellulose, increasing cost and complexity of bioethanol production from cellulosic biomass [4].

Recent approaches to improving switchgrass biomass quality have focused on engineering genes involved in the lignin biosynthesis pathway. Switchgrass plants with down-regulated caffeic acid o-methyltransferase (COMT) evaluated in the field had biomass with 10 to 14% reduced lignin concentration, 34% greater sugar release and 28% higher ethanol yield compared to control plants [5]. Despite these results, there are administrative challenges to commercializing transgenic switchgrass due to the deregulation process [6]. Switchgrass pollen retains its viability for up to 60 min, 100 min in rare cases, and may travel up to 3.5 km under mild wind conditions [7]. As a native grass species with less than 1% self-compatibility, the existence of viable pollen over large distances will result in migration of transgenes into native grasslands [8]. Autoexcision was investigated as a solution for preventing transgene flow, resulting in reduction of transgene flow by about 22–24% [9]. Traditional plant breeding for improved biomass quality represents an alternative approach to reduce recalcitrance of switchgrass biomass [10, 11]. Switchgrass populations divergently selected for *in vitro* dry matter digestibility (IVDMD) in a livestock production system showed a strong genetic correlation between IVDMD and ethanol yield of r = 0.84 [12]. This strong and positive genetic correlation indicates that the genetic basis underlying improvements in IVDMD could point to opportunities to improve ethanol yield from switchgrass biomass.

Forward genetic screening for causal alleles underlying the phenotypic variations in the natural populations can be carried out in light of high resolution of single nucleotide polymorphisms (SNPs) [13]. Different methodologies were applied depending on the populations under investigation. Allele segregation patterns were used to indicate causal markers in crossing populations, while the association between the genetic variance and the phenotypic variance was used in linkage disequilibrium mapping. Detection of allele frequency (AF) changes has been implemented in studying adaptively or artificially divergent populations [14–17]. Considering the large sample size needed to account for high density genetic variances in the natural populations, bulking the extremely divergent samples could drastically reduce the genotyping cost, and have been exploited successfully to detect SNPs associated with phenotype divergence [18, 19].

Numerous gene members in the monolignol biosynthesis pathway can affect lignin concentrations and forage digestibility in various species. COMT catalyzing methylation in the monolignol pathway [20, 21] is critical for the formation of two of the three monolignol units, guaiacyl (G) and syringyl (S). Down-regulation of the COMT2 gene in switchgrass reduced lignin concentration, S/G ratio and the recalcitrance to the fermentation process [22] indicating its methylation function in switchgrass lignin biosynthesis. The cinamyl alcohol dehydrogenase (CAD) gene catalyzes the reduction of hydoxycinnamyl aldehydes into their corresponding alcohols in the last steps of the monolignol pathway [23]. The class I CAD, also known as *bona fide* CAD, is hypothesized to be correlated with the origin of lignin, based on evidence from phylogenetic distance of CAD genes and the lack of lignin found in the earliest plants without *bona fide* CAD genes [24]. The CAD2 gene found in switchgrass is close to ZmCAD2 in maize and OsCAD2 in rice which were also classified as *bona fide* CAD [24]. Genetic engineering studies in alfalfa, rice, and forage grasses also demonstrated the influence of various monolignol genes on lignin concentration and degradation [25–27]. Genetic engineering of hydroxycinnamoyl-CoA shikimate/quinate hydroxycinnamoyl transferase (HCT) in alfalfa and COMT and CAD genes in tall fescue resulted in disrupted lignin biosynthesis. In switchgrass, transgenenic plants were generated by downregulating monolignol genes encoding COMT [22], CAD [28, 29], and 4-coumarate: CoA ligase 1 (4CL1) [30], each showing a significant decrease in lignin concentration and increases in ethanol production efficiency. The extensive forward and reverse genetic studies on these monolignol genes made them good candidate genes to investigate the genetic mechanisms underlying switchgrass breeding populations divergent in lignin concentrations.

The switchgrass populations used in this study were generated by divergent breeding for decreased and increased in vitro dry matter digestibility (IVDMD) at the USDA-ARS grass breeding project at the University of Nebraska-Lincoln, Nebraska [12]. The IVDMD test simulates the digestion of forages or biomass in ruminants. Five divergent populations in this study was generated from the selection process, including one base population (C0), the population of one selection cycle for low IVDMD (C-1), and the populations of five selection cycles for high IVDMD (C+1 to C+3) [12]. Lignin concentration, IVDMD and ethanol yield across the divergent populations changed substantially due to selection (S1 Fig). While IVDMD increased by 9.6% from population C-1 to C+3, acid detergent lignin (ADL) decreased by 17%, and ethanol yield increased by 12.7% [12]. The recurrent selection cycles resulted in significant differences and consistent rankings of IVDMD, ethanol yield and ADL values across the selection cycles [12, 31].

To identify polymorphisms responsible for the distinct differences of IVDMD, lignin concentration and ethanol yield in the breeding populations, candidate genes in the monolignol biosynthesis pathway were investigated in this study. We described the approach of detecting selection signatures in divergent selected switchgrass populations by AF changes. The identifications of polymorphisms under selection provided insight into the genetic basis of recurrent selection in switchgrass, and potential SNP markers to facilitate marker-assisted selection.

## Materials and Methods

### Plant materials

A random sample of five generations of divergent selection for IVDMD (populations C-1 through C+3) was space-transplanted in the field in May 2006. The populations are described as NE Trailblazer C-1, NE Trailblazer C0, Trailblazer, NE Trailblazer C2, and NE Trailblazer C3 in the official release notification by USDA-ARS. For our purposes, Trailblazer is noted as C+1 and the other four populations are noted according to their cycle number from the original population (C-1, C0, C+2, and C+3). The sign refers to the direction of selection for IVDMD and the number refers to the number of selection cycles or recombination events. The breeding generation evaluation nursery was established in 2006 with a randomized complete block design in Lincoln, Nebraska [12]. Within each of the six blocks, ten individual genotypes from each breeding generation were planted in a plot. Leaf samples were collected from each individual plant, freeze-dried, and sent to Madison, Wisconsin in 2010.

### Gene sequencing and genetic diversity

The dried leaf samples of five populations were pooled by population with 0.002g per individual. DNA extractions were made for each pool using the protocol described by [32]. Candidate genes COMT1, COMT2, CAD2, and 4CL1 were amplified from the genomic DNA using the NCBI cDNA sequences and primers shown in S1 Table. A high fidelity polymerase and minimum number of PCA cycles were used in genomic amplification to reduce PCA errors. Due to the high heterozygosity levels in switchgrass, the amplicons were cloned and sequenced individually by Sanger Sequencer. Middle primers were designed to sequence each amplicon as a haplotype read. A sample of sequences was obtained from each population pool for each of the four candidate genes (S2 Table). About 190 reads were sequenced from the C0 population pool to increase the precision of initial AF estimation in the AF tests.

To control the sequence quality, preliminary AF was calculated for each polymorphic site as the proportion of the minor polymorphism to the total reads across all five populations. The polymorphic sites with preliminary AFs lower than 0.05 were discarded. If a site has more than one minor allele and one of the preliminary AFs was greater than 0.05, the site cannot be discarded, but the haplotypes corresponding to the rare polymorphisms at this site were discarded. The AF per site per population were then calculated to be used in the statistical tests for selection signature.

Nucleotide diversity and haplotype diversity were calculated using DNaSP [33, 34]. Nucleotide diversity (π) was calculated as the average number of nucleotide differences per site between two sequences [35]. Pairwise linkage disequilibrium (LD) were estimated as r^2^ for both SNPs and InDels in R 3.2.0 [36]. LD values were fitted in the nonlinear models against the distances between pairs of SNPs. The distance of half decay LD is the distance when the predicted LD is half of its maximum value. The genetic diversity was also estimated within each population.

### Statistical tests for selection signature

The sequence data set were separated into the five divergent populations (C0, C-1, C+1-C+3), and the AFs were calculated within each population. The allele frequencies in the C0 population are the initial frequencies. Only the loci common among all 5 populations were kept for the following statistic tests.

The demographic scheme was simulated 10000 times with genetic drift only to build the null distributions for the statistic tests [37]. For each time of simulation, a base C0 population was generated using the initial AF observed in the real data. Each individual had a single diallelic polymorphic locus. Each locus was assigned eight alleles, which were randomly separated into two subgenomes during intercrossing, and followed a tetrasomic inheritance pattern within the subgenomes [38]. Individuals were randomly selected from the C0 population and producing progenies by polycrossing. The experimental error of estimating population AFs came from multiple sources at different levels, for example, the sampling error during population pooling, PCR amplification and clone picking. To account for these variations as much as possible, the sampling process was included in the simulation to calculate the simulated AF in each population. After simulation of each population, sixty individuals were randomly chosen to form an allele pool, and a number of alleles were randomly drawn according to the real number of reads obtained for each population pool. The simulated AF was calculated using this allele sample.

In the AF change test, thresholds were decided using the null distribution unique to each initial AF. The p-values were calculated as the proportion of simulated allele AF excessing the observed AF in a total of 10,000 simulations. The polymorphisms significant for the AF change test were then processed through a second, independent, statistical test: linear regression of AF on the cycle numbers. The slopes of the linear regression for each locus were compared to slopes generated from the simulated distribution. The p-values of both tests were adjusted by the controlled false discovery rate [39]. Selection signatures were considered to be significant only for loci that passed both tests with p-value <0.05.

## Results

### Gene sequencing and SNP discovery

Two family members of COMT (COMT1 and COMT2) and CAD2 and 4CL1 were sequenced using pooled genomic DNA from each population. The cDNA sequences of COMT2, CAD2, and 4CL1 in switchgrass were obtained from NCBI (accessions: HQ645965.1, GU045612.1, EU491511.1) [22, 30, 40]. Multiple members of COMT gene family were found by expression study in maize [41]. The coding sequence of COMT1 gene in switchgrass was identified by querying the most commonly expressed COMT member in maize by BLAST in NCBI EST database (accessions: FL749574.1, FL749575.1). These coding sequences were used for primer designs to amplify genomic sequences of the four genes. The measures including designing primer for specificity, using high fidelity polymerase and gel excision of the PCR amplicons were taken to make sure the amplification of interested members of the gene families in switchgrass.

**Fig 1:**
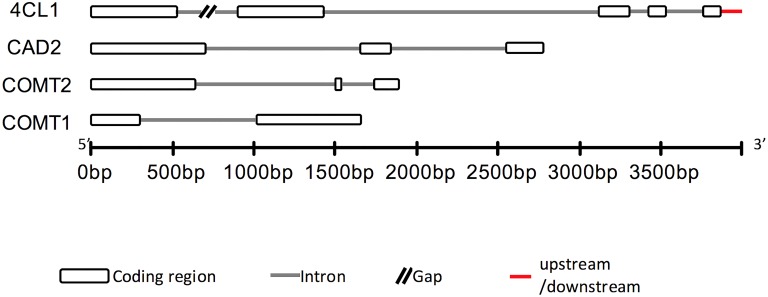
Hypothesized gene structures of the sequenced COMT1, COMT2, CAD2, and 4CL1 in switchgrass.

Gene structures of the resulted genomic sequences were inferred by comparing them to homologs from maize and sorghum (Fig 1). Thirteen exon regions and nine intron regions were sequenced. A small region of 3’ UTR was sequenced in 4CL1. The SNP markers were discovered by aligning the sequences from all five populations. To control the errors from PCR amplification and sequencing, polymorphic sites with preliminary AF less than 0.05 were discarded. As a result of the quality control, all the polymorphic sites were biallelic. The NCBI accession numbers of the aligned sequences are KY004561-KY004928 for COMT1, KY004196-KY004560 for COMT2, KY005440-KY005851 for CAD2 and KY004929-KY005439 for 4CL1.

The number of SNPs and InDels among all five populations were summarized in Table 1. A total of 183 SNPs and InDels were identified. The number of SNPs per 100 bp ranged from 0.83 for 4CL1 to 1.73 for COMT1. SNPs in the coding regions were found for all four genes, of which 17 are synonymous and 7 are nonsynonymous. No InDel was found in the coding regions. The intron regions have a total of 159 polymorphisms, of which 101 sites are SNPs, and 58 sites are InDels. No polymorphic sites in the 3’ UTR of 4CL1 gene was found.

**Table 1:** Total number of polymorphisms for the four candidate genes in switchgrass divergent populations.

	Whole gene	non-coding regions	coding regions	synonymous	non-synonymous
COMT1					
Sequence length(bp)	1851	978	873	NA	NA
# of SNP sites	32	29	3	3	0
# of Insert/Delete sites	34	34	0	0	0
SNP sites per 100 bp	1.73	2.97	0.34	NA	NA
COMT2					
Sequence length (bp)	1580	683	897	NA	NA
# of SNP sites	27	19	8	6	2
# of Insert/Delete sites	3	3	0	0	0
SNP sites per 100 bp	1.71	2.78	0.89	NA	NA
CAD2					
Sequence length (bp)	2789	2165	624	NA	NA
# of SNP sites	39	31	8	6	2
# of Insert/Delete sites	11	11	0	0	0
SNP sites per 100 bp	1.40	1.43	1.28	NA	NA
4CL1					
Sequence length (bp)	3255	2271	984	NA	NA
# of SNP sites	27	22	5	2	3
# of Insert/Delete sites	10	10	0	0	0
SNP sites per 100 bp	0.83	0.97	0.51	NA	NA

### Genetic diversity and linkage disequilibrium of polymorphisms in four candidate genes

**Fig 2:**
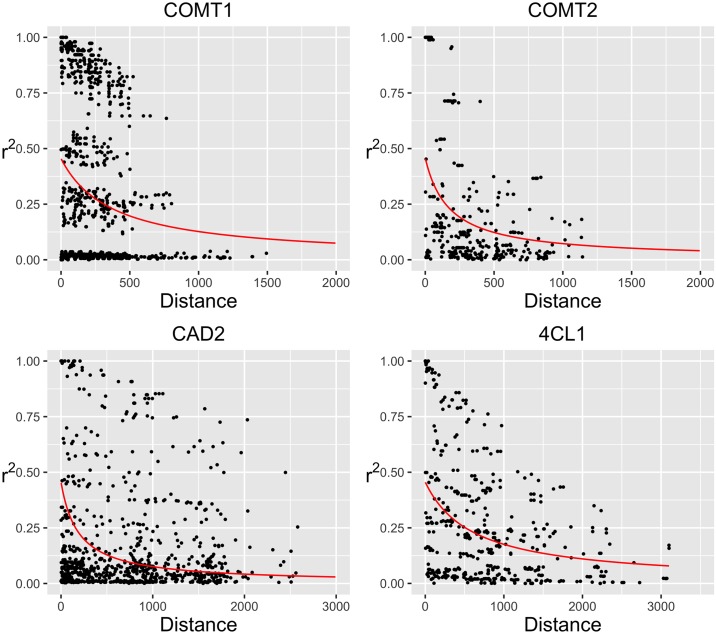
The scatter plots of pairwise LD on the distances between the polymorphic sites. The red line noted the predicted LD by fitted a nonlinear model of LD.

**Fig 3:**
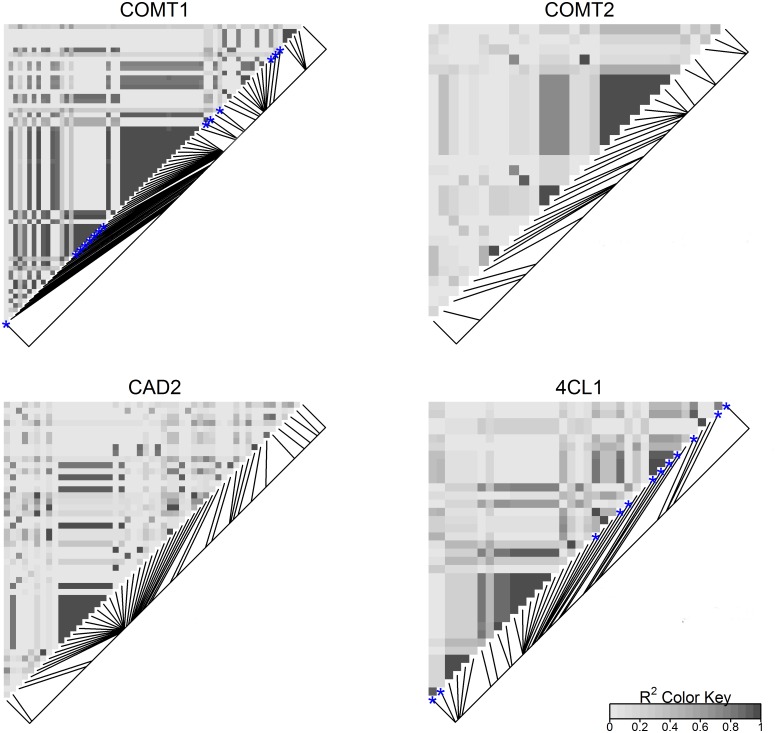
The heatmaps of pairwise LD of the polymorphisms in the four candidate genes. The blue stars indicate the significant loci under selection.

Nucleotide diversity was estimated for each gene ranging from 0.0027 to 0.0060 (Table 2). 4CL1 has the lowest overall diversity amongst the four genes. The diversity of synonymous sites is only slightly lower than the overall gene diversity. The ratio of diversity between synonymous sites and nonsynonymous sites were 7.8, 6.0 and 1.4 for CAD2, COMT2, and 4CL1 respectively. Haplotype diversity and LD were analyzed for each gene across all populations. A considerable amount of haplotypes was found for each gene, from 47 haplotypes in COMT2 to 100 in 4CL1, increasing as the lengths of the gene sequences increased. The rank of haplotype diversity for the genes differed from the number of haplotypes. 4CL1 has the highest number of haplotypes and medium level of Haplotype diversity 0.80. In the contrary, COMT2 has the lowest number of haplotypes and the highest haplotype diversity 0.93. Many of the haplotypes were represented by only one read each. The common haplotypes for each gene are the ones that have more than 5% reads. The number of the common haplotypes are drastically reduced and differed from 4 to 8 for the four genes. As expected, LD decayed rapidly along the genes. The overall means of pairwise LD (r2) were lower than 0.4. LD reduced to half within only several hundred base pairs for all of the genes (Fig 2). The mosaic patterns in the LD heatmaps in (Fig 3) showed very short LD blocks at the candidate genes in the octoploid switchgrass populations.

**Table 2:** Genetic diversity and LD in each of the four candidate genes. Different nucleotide diversity was estimated using SNPs within the whole gene, π, nonsynonymous SNP sites, π(nonsyn), synonymous SNP sites, π(syn), and the silent SNP sites including both synonymous and non-coding sites, π(s). The results of haplotype and LD analysis include number of haplotypes (H), haplotype diversities (Hd), the number of haplotypes with proportions higher than 0.05 (H>0.05), mean of pairwise LD (LD mean) and the half LD decay distance (LD decay).

Gene	π	π (nonsyn)	π(syn)	π(s)	H	Hd	H (>0.05)	LD mean	LD decay (bp)
**COMT1**	0.0043	0.0000	0.0039	0.0067	53	0.741	5	0.334	375
**COMT2**	0.0060	0.0011	0.0068	0.0096	47	0.927	8	0.224	188
**CAD2**	0.0043	0.0014	0.0109	0.00486	87	0.920	5	0.196	204
**4CL1**	0.0027	0.0015	0.0021	0.0031	100	0.803	4	0.258	609

The phylogenetic tree of the haplotypes indicated that despite the number of haplotypes discovered, the haplotypes within each gene have no significant branching. The substituted amino acids were analyzed for the impact of substitution on protein function in SIFT [42]. There was no significant predicted impact on protein function for all of the non-synonymous loci.

### Allele frequencies in the extreme cycles for four candidate genes

To investigate the association of polymorphisms in the four genes with selection for IVDMD, allele frequencies and AF changes between the most extreme populations were analyzed. Minor allele frequencies of the SNPs/InDels were calculated within each population pool. The AF changes were calculated as the AF in C+3 population (high IVIDMD) minus the AF in C-1 population (low IVDMD).

**Fig 4:**
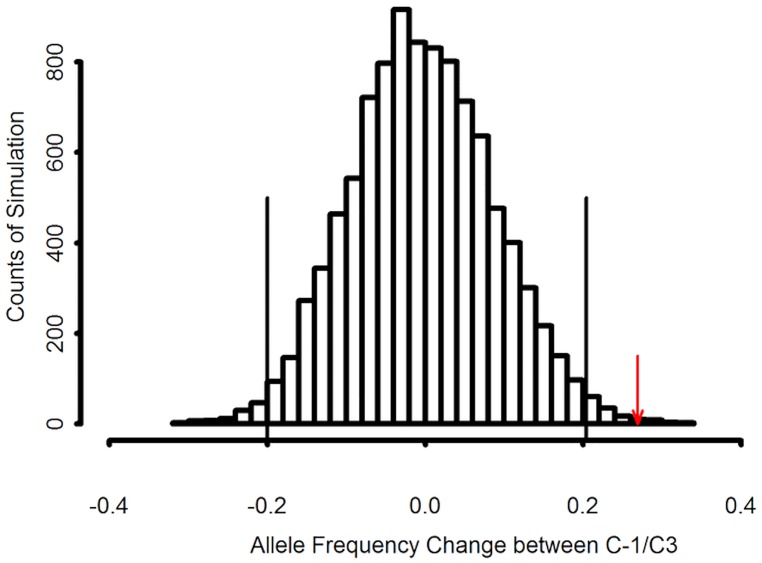
Distribution of simulated allele frequency change between C-1 and C+3 for an initial allele frequency in C0 of 0.15 at locus 246 of COMT1 gene. The red arrow indicates the observed change in allele frequency between cycles C-1 and C+3. The lines indicate the Benjamini-Hochberg-adjusted confidence intervals of allele frequency change with one-tailed test with α = 0.05.

During short-term selections, two reasons could result in AF changes across the genome, genetic drift and selection, one causing random AF fluctuation, while the other producing directional frequency changes. The loci under constant selection would likely have bigger AF changes than the loci undergone only genetic drift, if the selection intensity is high or the trait under selection is highly inheritable. A demographic scheme was simulated (S2 Fig) to reflect the population size changes from generation to generation in the IVDMD breeding project, except that random individuals got to pass their alleles down to the next generation. This is the genetic drift effect that would occur on the neutral loci during the breeding process. A distribution of the AF changes at a certain locus was obtained by repeatedly simulating the demographic process for 10,000 times. This distribution provided the null distribution for the hypothesis that there is no selection effect at a locus, only genetic drift. Therefore, the loci with AF changes exceeding the thresholds defined by the simulated distribution were determined to be under selection (Fig 4). The significant levels were calculated in the one-tailed statistic tests as the ratio between the number of simulations with bigger/smaller AF changes than the observed AF change and the total 10,000 simulations.

**Fig 5:**
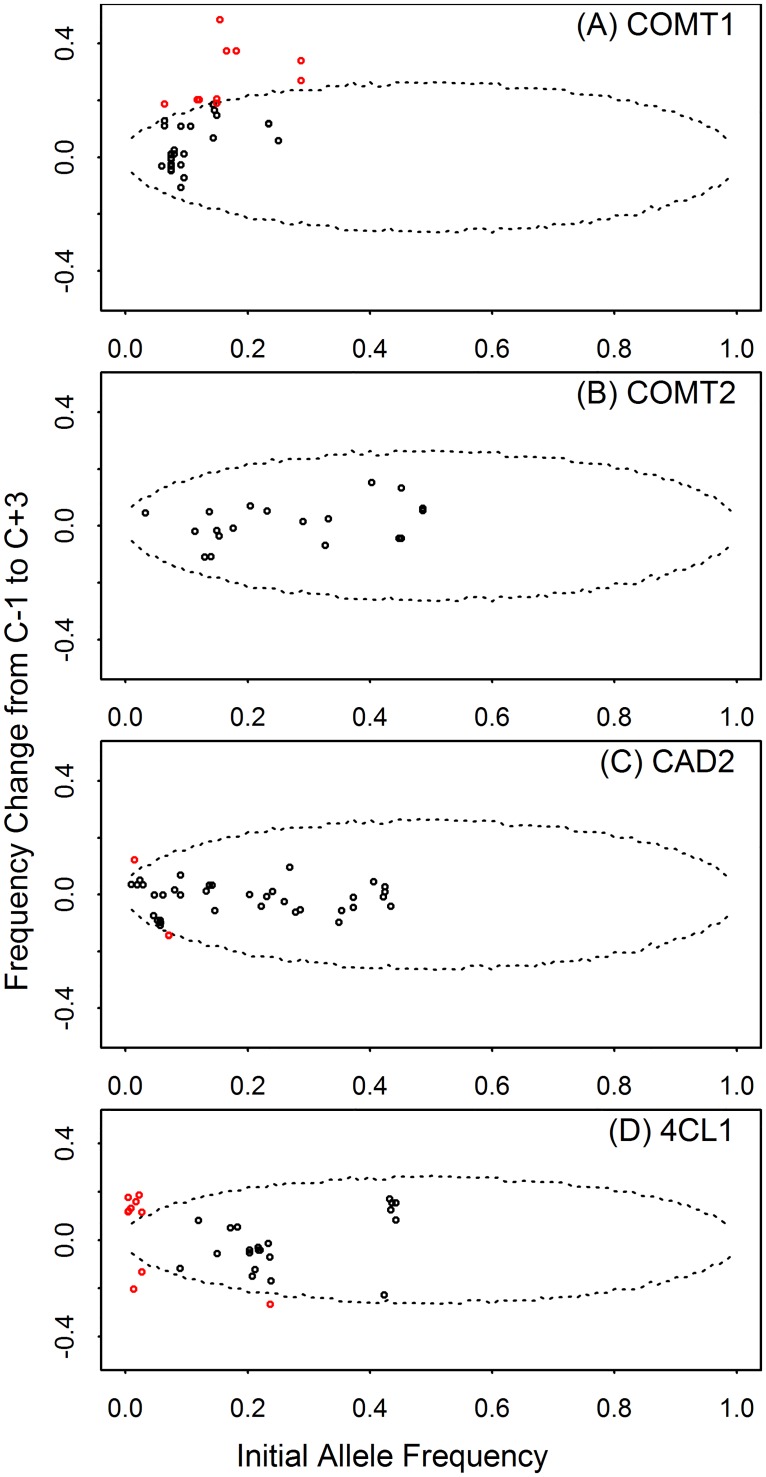
Changes in allele frequency between divergent breeding populations C-1 and C+3 for COMT1, COMT2, CAD2 and 4CL1 in switchgrass. The data points are observed allele frequency changes plotted on the initial allele frequencies from C0. The dotted lines indicated the Benjamini-Hochberg-adjusted confidence intervals (CI) (α = 0.05) of allele frequency changes using the 10,000-simulation data. Data points inside the CI are deemed due to drift, while those outside the CI (shown in red color) are deemed candidates for selection.

In total, 36 SNPs and InDels were found significant for AF changes after adjusting p-values to control FDR (Fig 5). None of the nonsynonymous sites were found significant. Out of the 37 polymorphisms, 25 of them located in the intron regions, and 3 are synonymous polymorphisms in the exon regions. Ranges of AF changes for all the observed SNPs/InDels were: -0.11 to 0.48 for COMT1, -0.11 to 0.15 for COMT2, -0.15 to 0.12 for CAD2, and -0.26 to 0.18 for 4CL1. The COMT1 gene had the widest range of AF change among the four genes, followed by 4CL1. Due to the AF changes at the significant loci, rare alleles were turned into frequent or common alleles at the end of the selection cycle, and *vice versa*. Even though CAD2 and COMT2 had medium levels of genetic diversity comparing, they have fewer loci with significant AF changes.

### Linear regression of allele frequencies against selection cycles

The SNPs/InDels significant in the AF change test were analyzed by regression of allele frequencies on selection cycles. Slopes of the linear regression in the observed data were compared with that calculated in the simulated data to determine p-values. Eighty percent of the significant polymorphisms from the AF change test were also significant in the regression test. As a result, 29 SNPs and InDels passed both tests as final significant polymorphisms associated with recurrent selection. All 7 polymorphisms that didn’t pass the regression coefficient test were from CAD2 gene. Significant loci were detected only in COMT1 and 4CL1.

**Fig 6:**
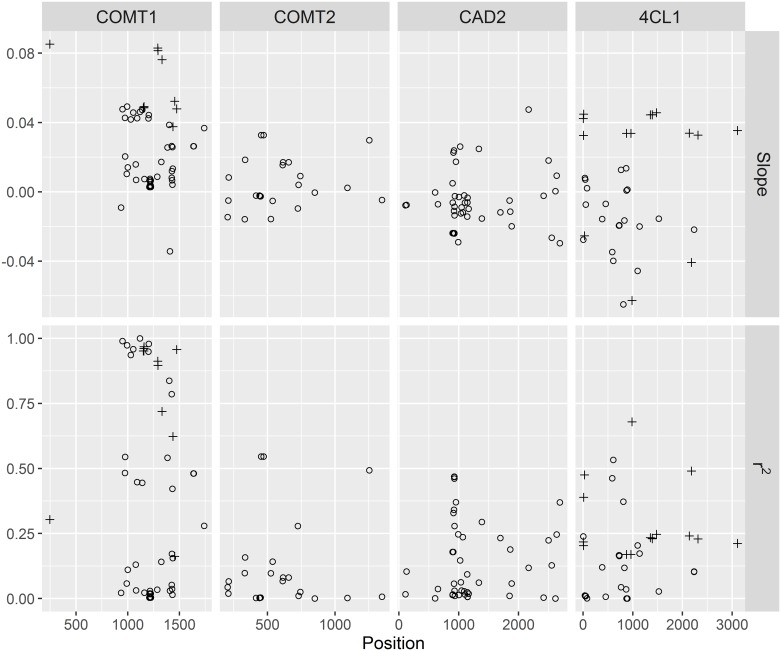
Slope (change in allele frequency per cycle of selection) and fit of the linear regressions (r^2^) for the polymorphisms with significant allele frequency change across the selection cycles. Plus signs represent polymorphisms with P≤0.05, and open circles represent polymorphisms with P>0.05.

The fit of the linear regression (r^2^) and the slopes (b) were plotted against their physical positions in each gene (Fig 6). The b values of the significant loci clustered together as the LD blocks in COMT1 and 4CL1. For the significant polymorphisms, the average of absolute b values is 0.058 in COMT1, and 0.040 in 4CL1. The sign of b is the direction of AF change of the minor alleles at significant loci, with a positive sign indicating that the minor allele. All of the significant loci of COMT1 have positive b values, indicating that the minor alleles at these loci have positive effects on IVDMD and negative impact on lignin concentration. In 4CL1 the significant b values had mixed signs for the significant loci within the range of -0.063 to 0.046. The loci with positive b in 4CL1 were intervened by loci with negative b, which corresponded to the pairwise LD patterns in 4CL1 (Fig 3). The synonymous SNPs in the coding regions have b values of 0.085 and 0.048 in COMT1 and 0.035 and 0.042 in 4CL1. The linear regression of synonymous SNPs has goodness-of-fit values ranging from 0.21 in 4CL1 to 0.96 in COMT1.

### Allele frequency, genetic diversity and haplotypes change across the selection cycles

**Fig 7:**
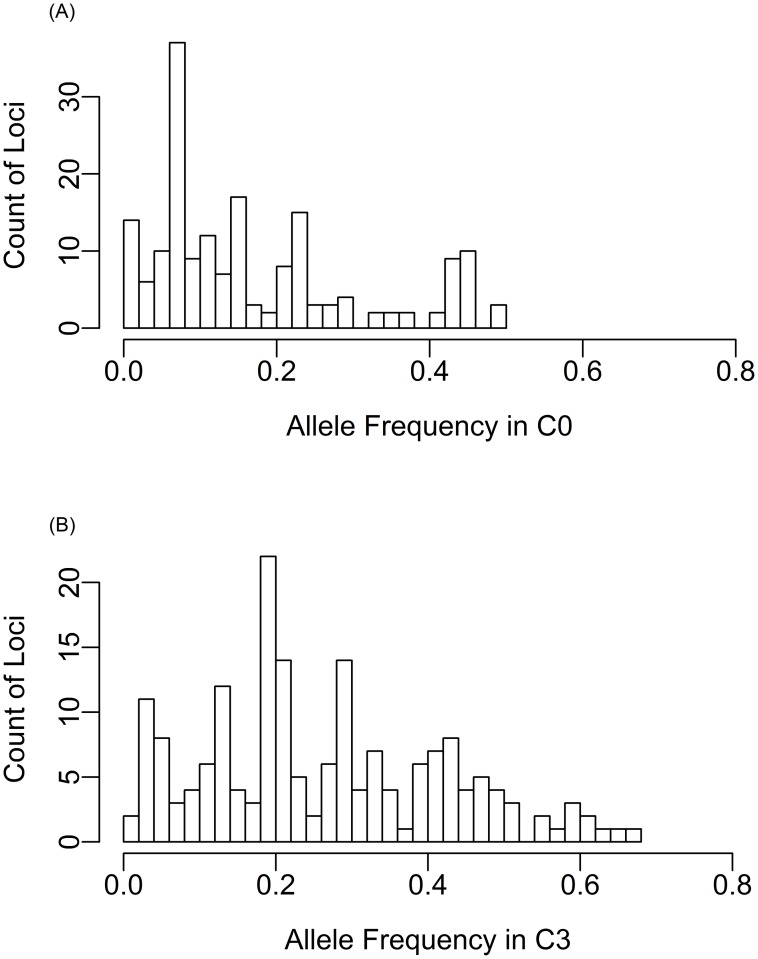
Histograms of allele frequency on all 183 polymorphisms undergone statistic tests in C0 and C+3 populations.

Enriched intermediate-frequency alleles and a slight increase of genetic diversity are observed as expected for the positive selection on standing variation during a short term [43]. The AF spectrum of all 180 polymorphic loci in population C0 and C+3 were plotted in histograms (Fig 7). The C0 population has enriched number of loci with low frequency alleles and very low counts of loci having allele frequencies higher than 0.3. After three cycles of selection, the allele frequencies distribution shifted distinctively, resulting in an increased number of alleles with intermediate frequencies ranging from 0.2 to 0.5 and decreased number of alleles in the range of 0 to 0.20. Genetic diversity and haplotype diversity of each gene was calculated for each selection cycle. Within COMT1 and 4CL1 where significant polymorphisms were found, the nucleotide diversity (π) increased in both directions after one cycle of divergent selection, and continued to increase as the selection cycles increased (S3 Table). While in COMT2 and CAD2 genes, no clear trend was seen. The increase of the moderate AF also coincided with the increase of π in COMT1 and CAD2.

## Discussion

Switchgrass produces a high yield of lignocellulosic biomass, especially with recent advances in breeding for increased biomass production [44]. Reducing recalcitrance of switchgrass biomass to fermentation has been a long-term research objective toward improving the economics and sustainability of livestock production [45]. Parallels between ruminant livestock fermentation and biomass fermentation for bioethanol suggest similar mechanisms for biomass recalcitrance [12, 46]. The divergent populations generated from recurrent selected for IVDMD [31] provided powerful tools to identify the polymorphisms under selection and the candidate polymorphisms associated with lignin concentration and ethanol yield. Existence of discreet selection cycles and the availability of genotypes from all the intermediate cycles facilitated detection of selection signatures using both an allele divergence test and a linear regression test.

Multiple polymorphisms in the candidate genes were found under selection for IVDMD. Artificial selection has been known to affect a number of genomic regions for traits such as ear number [16], seed size [17], and disease resistance [47] in maize long-term breeding populations. Multiple genes involved in the monolignol pathway were also found associated with digestibility traits in the maize breeding lines [48]. Similar results were observed in other association studies in maize [48–53], sorghum [54], alfalfa [55] and perennial ryegrass [56–58]. The bigger b values in COMT1 suggested that larger phenotypic changes associated with selection on these polymorphisms than the polymorphisms in 4CL1 [59].

Complex traits like IVDMD are controlled by multiple loci with small effects [49]. The anatomic study in the divergent genotypes of these breeding populations showed reduced lignification, fewer cortical sclerenchyma in the stem tissues and more parenchyma cells in some vascular bundles, which indicated that besides lignin biosynthesis, other pathways affecting cell development could also be selected while breeding for divergent IVDMD [60, 61]. In this study, we chose to investigate four candidate genes in three functionally characterized gene families in switchgrass [22, 28, 29, 30]. None of the non-synonymous polymorphisms within the sequenced candidate genes was significant, suggesting that the significant polymorphisms could be involved in trans-regulation, or the causal genes could be in LD with COMT1 and 4CL1. Genome-wide molecular markers are needed to gain a complete picture of genetic controls of IVDMD in these short-term breeding populations.

Different monolignol genes in these divergent switchgrass populations showed low to medium nucleotide diversities. Nucleotide diversity of COMT genes in this study fell within the similar range as the estimations in maize (*Zea mays*) and alfalfa (*Medicago sativa*) [55, 62, 63]. The 4CL1 gene had lower diversity level than that in the maize inbred lines. The nucleotide diversity of resistance genes in diverse switchgrass populations ranged from 0.0051 to 0.072, slightly higher than the estimations in this study [64]. Different regions of the genome and the population origins could contribute to the relatively low nucleotide diversity [65].

The patterns of genetic variation in these candidate genes depicted the complexity of the octoploid switchgrass genomes. Majority of the significant loci have initial allele frequencies in the low range (<0.2) except for two loci in the COMT1 gene. This could be explained by that genetic diversity for low lignin and high IVDMD traits are not necessary for surviving in the wild habitat, sometimes might even be defective [66]. Before selection, the alleles beneficial for bioethanol production could arise by mutation and preserved in the genome by many different haplotypes each at a relatively low frequency resulting in a relatively low nucleotide diversity. The selection force accumulated the beneficial alleles and the haplotypes that harbor these alleles, which resulted in low LD within a gene. It is interesting to note that 4CL1 gene have mixed signs of b, and lower LD while the COMT1 gene has much more defined and longer LD blocks. Giving the unstable nature of chromosome pairing and segregating in polyploid switchgrass, recombination could be indirectly selected during the breeding process, even within a short term [67, 68], which could explain that both directions of selection were observed in 4CL1 gene. The genetic patterns revealed in the breeding populations suggested the need of developing a comprehensive selection criteria or germplasm pool to maintain the overall performance of the breeding populations especially for long term selection.

The significant polymorphisms discovered in this study are potential candidates for QTL underlying biomass quality in switchgrass, and provided possible markers for marker-assisted selection [69, 70]. Depending on the number of QTL, heritability of the traits and genomic models, genomic selection could also increase the favorable allele frequencies of QTL at various rates [71]. The number of significant polymorphisms suggested that the individual loci underlying recalcitrance to biomass conversion had small effects, which was also observed in the maize cell wall component traits [72]. However, the low LD in the switchgrass promises the potential of genetic gain under the appropriate selection scheme. To effectively improve biomass quality in switchgrass, breeding projects could benefit greatly from the marker-assisted selection by increasing the favorable alleles of the QTL recurrently.

## Supporting Information

S1 Fig Changes in in vitro dry matter digestibility (IVDMD), ethanol production and lignin concentration across the five populations evaluated in Lincoln, Nebraska. The figure is adapted from data of Vogel and others [12] for illustrative purpose only, not a replicate of published images.
(TIF)

S2 Fig Population sizes through divergent recurrent selection for in vitro dry matter digestibility in switchgrass. From the base population C0, one cycle of selection for low IVDMD and three cycles of selection for high IVDMD were conducted, resulting in four selected populations, C-1, C+1, C+2 and C+3. Population sizes are represented by n and the number of selected individuals by m for each group of selected individuals (S-1, S+1, S+2, and S+3). The figure is adapted from data of Vogel and others [12] for illustrative purpose only, not a replicate of published images.
(TIFF)

S1 Table Summary information on allele sequences for four candidate genes obtained from the five divergent populations. Switchgrass v3.1 genomic identifier were obtained from phytozome genome database by using our sequences as queries in BLAST.
(DOCX)

S2 Table The number of gene sequences sampled from each population allele pool. (DOCX)

S3 Table Genetic diversity and haplotype diversity within the divergent populations for the four candidate genes. (DOCX)

## References

[1] EIA. Biofuels production drives growth in overall biomass energy use over past decade http://www.eia.gov/todayinenergy/detail.cfm?id=15451-2014 [cited 2014 March 18].

[2] Wang M, Han J, Dunn JB, Cai H, Elgowainy A. Well-to-wheels energy use and greenhouse gas emissions of ethanol from corn, sugarcane and cellulosic biomass for US use. Environmental Research Letters. 2012;7(4). 10.1088/1748-9326/7/4/045905. WOS:000312696400077.

[3] Schmer MR, Vogel KP, Mitchell RB, Perrin RK. Net energy of cellulosic ethanol from switchgrass. Proceedings of the National Academy of Sciences of the United States of America. 2008;105(2):464–9. 10.1073/pnas.0704767105. WOS:000252551100015. 18180449 PMC2206559

[4] Banerjee S, Mudliar S, Sen R, Giri B, Satpute D, Chakrabarti T, et al Commercializing lignocellulosic bioethanol: technology bottlenecks and possible remedies. Biofuels Bioproducts & Biorefining-Biofpr. 2010;4(1):77–93. 10.1002/bbb.188. WOS:000274525300018.

[5] Baxter HL, Mazarei M, Labbe N, Kline LM, Cheng Q, Windham MT, et al Two-year field analysis of reduced recalcitrance transgenic switchgrass. Plant Biotechnology Journal. 2014;12(7):914–24. 10.1111/pbi.12195. WOS:000340528000010. 24751162

[6] Wang Z-Y, Brummer EC. Is genetic engineering ever going to take off in forage, turf and bioenergy crop breeding? Annals of Botany. 2012;110(6):1317–25. 10.1093/aob/mcs027. WOS:000310371800022. 22378838 PMC3478041

[7] Ge Y, Fu C, Bhandari H, Bouton J, Brummer C, Wang ZY. Pollen viability and longevity of switchgrass (*Panicum virgatum* L.). In Vitro Cellular & Developmental Biology-Plant. 2012;48(4):430-. WOS:000308227200009.

[8] Liu LL, Thames SYL, Wu YQ. Lowland switchgrass plants in populations set completely outcrossed seeds under field conditions as assessed with SSR markers. Bioenergy Research. 2014;7(1):253–9. 10.1007/s12155-013-9367-7. WOS:000332484000021.

[9] Somleva MN, Xu CA, Ryan KP, Thilmony R, Peoples O, Snell KD, et al Transgene autoexcision in switchgrass pollen mediated by the Bxb1 recombinase. Bmc Biotechnology. 2014;14 10.1186/1472-6750-14-79. WOS:000340908500001. 25148894 PMC4148497

[10] Godshalk EB, Burns JC, Timothy DH. Selection for in vitro dry-matter disappearance in switchgrass regrowth. Crop Science. 1986;26(5):943–7. WOS:A1986D826900021.

[11] Vogel KP, Haskins FA, Gorz HJ. Divergent selection for in vitro dry-matter digestibility in switchgrass. Crop Science. 1981;21(1):39–41. WOS:A1981LL16500011.

[12] Vogel KP, Mitchell RB, Sarath G, Jung HG, Dien BS, Casler MD. Switchgrass biomass composition altered by six generations of divergent breeding for digestibility. Crop Science. 2013;53(3):853–62. 10.2135/cropsci2012.09.0542. WOS:000319527000014.

[13] Schneeberger K. Using next-generation sequencing to isolate mutant genes from forward genetic screens. Nature Reviews Genetics. 2014;15(10):662–76. 10.1038/nrg3745. WOS:000342249700011. 25139187

[14] Turner TL, Bourne EC, Von Wettberg EJ, Hu TT, Nuzhdin SV. Population resequencing reveals local adaptation of *Arabidopsis lyrata* to serpentine soils. Nature Genetics. 2010;42(3):260–U42. 10.1038/ng.515. WOS:000274912400017. 20101244

[15] Flori L, Fritz S, Jaffrezic F, Boussaha M, Gut I, Heath S, et al The genome response to artificial selection: a case study in dairy cattle. Plos One. 2009;4(8). 10.1371/journal.pone.0006595. WOS:000268935900010. 19672461 PMC2722727

[16] Beissinger TM, Hirsch CN, Vaillancourt B, Deshpande S, Barry K, Buell CR, et al A genome-wide scan for evidence of selection in a maize population under long-term artificial selection for ear number. Genetics. 2014;196(3):829-+. 10.1534/genetics.113.160655. WOS:000333905500019. 24381334 PMC3948809

[17] Hirsch CN, Flint-Garcia SA, Beissinger TM, Eichten SR, Deshpande S, Barry K, et al Insights into the effects of long-term artificial selection on seed size in maize. Genetics. 2014;198(1):409–21. 10.1534/genetics.114.167155. MEDLINE:25037958. 25037958 PMC4174951

[18] Yang Z, Huang D, Tang W, Zheng Y, Liang K, Cutler AJ, et al Mapping of quantitative trait loci underlying cold tolerance in rice seedlings via high-throughput sequencing of pooled extremes. Plos One. 2013;8(7). 10.1371/journal.pone.0068433. WOS:000323114200009. 23935868 PMC3728330

[19] Ehrenreich IM, Torabi N, Jia Y, Kent J, Martis S, Shapiro JA, et al Dissection of genetically complex traits with extremely large pools of yeast segregants. Nature. 2010;464(7291):1039–U101. 10.1038/nature08923. WOS:000276635000038. 20393561 PMC2862354

[20] Parvathi K, Chen F, Guo DJ, Blount JW, Dixon RA. Substrate preferences of O-methyltransferases in alfalfa suggest new pathways for 3-O-methylation of monolignols. Plant Journal. 2001;25(2):193–202. WOS:000166980400007. 11169195 10.1046/j.1365-313x.2001.00956.x

[21] Naaz H, Pandey VP, Singh S, Dwivedi UN. Structurefunction analyses and molecular modeling of caffeic acid-O-methyltransferase and caffeoyl-CoA-O-methyltransferase: Revisiting the basis of alternate methylation pathways during monolignol biosynthesis. Biotechnology and Applied Biochemistry. 2013;60(2):170–89. 10.1002/bab.1075. WOS:000318044000005. 23600572

[22] Fu CX, Mielenz JR, Xiao XR, Ge YX, Hamilton CY, Rodriguez M, et al Genetic manipulation of lignin reduces recalcitrance and improves ethanol production from switchgrass. Proceedings of the National Academy of Sciences of the United States of America. 2011;108(9):3803–8. 10.1073/pnas.1100310108. WOS:000287844400066. 21321194 PMC3048149

[23] Anterola AM, Lewis NG. Trends in lignin modification: a comprehensive analysis of the effects of genetic manipulations/mutations on lignification and vascular integrity. Phytochemistry. 2002;61(3):221–94. WOS:000179017100002. 12359514 10.1016/s0031-9422(02)00211-x

[24] Guo DM, Ran JH, Wang XQ. Evolution of the cinnamyl/sinapyl alcohol dehydrogenase (CAD/SAD) gene family: the emergence of real lignin is associated with the origin of bona fide CAD. Journal of Molecular Evolution. 2010;71(3):202–18. 10.1007/s00239-010-9378-3. WOS:000281397000004. 20721545

[25] Chen F, Dixon RA. Lignin modification improves fermentable sugar yields for biofuel production. Nature Biotechnology. 2007;25(7):759–61. 10.1038/nbt1316. WOS:000247994000026. 17572667

[26] Chen L, Auh CK, Dowling P, Bell J, Chen F, Hopkins A, et al Improved forage digestibility of tall fescue (*Festuca arundinacea*) by transgenic down-regulation of cinnamyl alcohol dehydrogenase. Plant Biotechnology Journal. 2003;1(6):437–49. WOS:000188440200005. 17134402 10.1046/j.1467-7652.2003.00040.x

[27] Chen L, Auh CK, Dowling P, Bell J, Lehmann D, Wang ZY. Transgenic down-regulation of caffeic acid O-methyltransferase (COMT) led to improved digestibility in tall fescue (*Festuca arundinacea*). Functional Plant Biology. 2004;31(3):235–45. 10.1071/fp03254. WOS:000220831200004. 32688895

[28] Fu C, Xiao X, Xi Y, Ge Y, Chen F, Bouton J, et al Downregulation of cinnamyl alcohol dehydrogenase (CAD) leads to improved saccharification efficiency in switchgrass. Bioenergy Research. 2011;4(3):153–64. 10.1007/s12155-010-9109-z. WOS:000293018200001.

[29] Saathoff AJ, Sarath G, Chow EK, Dien BS, Tobias CM. Downregulation of cinnamyl-alcohol dehydrogenase in switchgrass by RNA silencing results in enhanced glucose release after cellulase treatment. PLoS One. 2011;6(1):e16416 10.1371/journal.pone.0016416 21298014 PMC3029337

[30] Xu B, Escamilla-Trevino LL, Sathitsuksanoh N, Shen Z, Shen H, Zhang YHP, et al Silencing of 4-coumarate:coenzyme A ligase in switchgrass leads to reduced lignin content and improved fermentable sugar yields for biofuel production. New Phytologist. 2011;192(3):611–25. 10.1111/j.1469-8137.2011.03830.x. WOS:000296850800009. 21790609

[31] Hopkins AA, Vogel KP, Moore KJ. Predicted and realized gains from selection for in vitro dry-matter digestibility and forage yield in switchgrass. Crop Science. 1993;33(2):253–8. WOS:A1993LC50800007.

[32] Edwards K, Johnstone C, Thompson C. A simple and rapid method for the preparation of plant genomic DNA for PCR analysis. Nucleic Acids Research. 1991;19(6):1349-. 10.1093/nar/19.6.1349. WOS:A1991FE01300035. 2030957 PMC333874

[33] Librado P, Rozas J. DnaSP v5: a software for comprehensive analysis of DNA polymorphism data. Bioinformatics. 2009;25(11):1451–2. 10.1093/bioinformatics/btp187. WOS:000266109500026. 19346325

[34] Nei M. Analysis of gene diversity in subdivided populations. Proceedings of the National Academy of Sciences of the United States of America. 1973;70(12):3321–3. 10.1073/pnas.70.12.3321. WOS:A1973R637400010. 4519626 PMC427228

[35] Masatoshi N. Molecular evolutionary genetics: Columbia University Press; 1987 512 p.

[36] Team RC. R: A language and environment for statistical computing R Foundation for Statistical Computing, Vienna, Austria 2015 Available from: http://www.r-project.org/.

[37] Waples RS. Temporal variation in allele frequencies—testing the right hypothesis. Evolution. 1989;43(6):1236–51. WOS:A1989AN91500007. 28564497 10.1111/j.1558-5646.1989.tb02571.x

[38] Triplett JK, Wang Y, Zhong J, Kellogg EA. Five nuclear loci resolve the polyploid history of switchgrass (*Panicum virgatum* L.) and relatives. Plos One. 2012;7(6). 10.1371/journal.pone.0038702. WOS:000305583300041. 22719924 PMC3377691

[39] Benjamini Y, Hochberg Y. Controlling the false discovery rate—a practical and powerful approach to multiple testing. Journal of the Royal Statistical Society Series B-Methodological. 1995;57(1):289–300. WOS:A1995QE45300017.

[40] Saathoff AJ, Sarath G, Chow EK, Dien BS, Tobias CM. Downregulation of cinnamyl-alcohol dehydrogenase in switchgrass by RNA silencing results in enhanced glucose release after cellulase treatment. Plos One. 2011;6(1). 10.1371/journal.pone.0016416. WOS:000286663900047. 21298014 PMC3029337

[41] Sekhon RS, Lin HN, Childs KL, Hansey CN, Buell CR, de Leon N, et al Genome-wide atlas of transcription during maize development. Plant Journal. 2011;66(4):553–63. 10.1111/j.1365-313X.2011.04527.x. WOS:000290456400001. 21299659

[42] Ng PC, Henikoff S. SIFT: predicting amino acid changes that affect protein function. Nucleic Acids Research. 2003;31(13):3812–4. 10.1093/nar/gkg509. WOS:000183832900117. 12824425 PMC168916

[43] Przeworski M, Coop G, Wall JD. The signature of positive selection on standing genetic variation. Evolution. 2005;59(11):2312–23. 10.1554/05-273.1. WOS:000233769000003. 16396172

[44] Casler MD, Vogel KP. Selection for biomass yield in upland, lowland, and hybrid switchgrass. Crop Science. 2014;54(2):626–36. 10.2135/cropsci2013.04.0239. WOS:000336746800019.

[45] Casler MD, Vogel KP. Accomplishments and impact from breeding for increased forage nutritional value. Crop Science. 1999;39(1):12–20. WOS:000078747300003.

[46] Han KJ, Pitman WD, Kim M, Day DF, Alison MW, McCormick ME, et al Ethanol production potential of sweet sorghum assessed using forage fiber analysis procedures. Global Change Biology Bioenergy. 2013;5(4):358–66. 10.1111/j.1757-1707.2012.01203.x. WOS:000319947300002.

[47] Wisser RJ, Murray SC, Kolkman JM, Ceballos H, Nelson RJ. Selection mapping of loci for quantitative disease resistance in a diverse maize population. Genetics. 2008;180(1):583–99. 10.1534/genetics.108.090118. WOS:000259758500047. 18723892 PMC2535707

[48] Andersen JR, Zein I, Wenzel G, Krutzfeldt B, Eder J, Ouzunova M, et al. Linkage disequilibrium and associations with forage quality at loci involved in monolignol biosynthesis in breeding lines of European silage maize (Zea mays L.). Proceedings of the XXVIIth EUCARPIA Symposium on improvement of fodder crops and amenity grasses, Copenhagen, Denmark, 19th to 23rd August 2007. 2007:145–9. CABI:20093194875.

[49] Truntzler M, Barriere Y, Sawkins MC, Lespinasse D, Betran J, Charcosset A, et al Meta-analysis of QTL involved in silage quality of maize and comparison with the position of candidate genes. Theoretical and Applied Genetics. 2010;121(8):1465–82. 10.1007/s00122-010-1402-x. WOS:000283501900007. 20658277

[50] Alarcon-Zuniga B, Hernandez-Garcia A, Vega-Vicente E, Cervantes-Martinez C, Warburton M, Cervantes-Martinez T. Genetic diversity and association mapping of three O-methyltransferase genes in maize and tropical grasses Molecular Breeding of Forage and Turf. 2009:151–62. 10.1007/978-0-387-79144-9_14. WOS:000261301000014.

[51] Brenner EA, Zein I, Chen YS, Andersen JR, Wenzel G, Ouzunova M, et al Polymorphisms in O-methyltransferase genes are associated with stover cell wall digestibility in European maize (*Zea mays* L.). Bmc Plant Biology. 2010;10 10.1186/1471-2229-10-27. WOS:000275403300001. 20152036 PMC2829591

[52] Chen YS, Zein I, Brenner EA, Andersen JR, Landbeck M, Ouzunova M, et al Polymorphisms in monolignol biosynthetic genes are associated with biomass yield and agronomic traits in European maize (*Zea mays* L.). Bmc Plant Biology. 2010;10 10.1186/1471-2229-10-12. WOS:000275401300001. 20078869 PMC2827421

[53] Andersen JR, Zein I, Wenzel G, Darnhofer B, Eder J, Ouzunova M, et al Characterization of phenylpropanoid pathway genes within European maize (*Zea mays* L.) inbreds. BMC Plant Biology. 2008;8(2):(03 January 2008)-(03 January). CABI:20083065537. 10.1186/1471-2229-8-2 PMC2265712 18173847

[54] Wang Y-H, Acharya A, Burrell AM, Klein RR, Klein PE, Hasenstein KH. Mapping and candidate genes associated with saccharification yield in sorghum. Genome. 2013;56(11):659–65. 10.1139/gen-2013-0134. WOS:000327944400003. 24299105

[55] Sakiroglu M, Sherman-Broyles S, Story A, Moore KJ, Doyle JJ, Brummer EC. Patterns of linkage disequilibrium and association mapping in diploid alfalfa (*M*. *sativa* L.). Theoretical and Applied Genetics. 2012;125(3):577–90. 10.1007/s00122-012-1854-2. WOS:000306432700013. 22476875 PMC3397135

[56] Pembleton LW, Wang J, Cogan NOI, Pryce JE, Ye G, Bandaranayake CK, et al Candidate gene-based association genetics analysis of herbage quality traits in perennial ryegrass (*Lolium perenne* L.). Crop & Pasture Science. 2013;64(3):244–53. 10.1071/cp12392. WOS:000322775500005.

[57] Cogan N, Smith K, Yamada T, Francki M, Vecchies A, Jones E, et al QTL analysis and comparative genomics of herbage quality traits in perennial ryegrass (*Lolium perenne* L.). Theoretical and Applied Genetics. 2005;110(2):364–80. 10.1007/s00122-004-1848-9. WOS:000226553900019. 15558228

[58] Cogan NOI, Ponting RC, Vecchies AC, Drayton MC, George J, Dracatos PM, et al Gene-associated single nucleotide polymorphism discovery in perennial ryegrass (*Lolium perenne* L.). Molecular Genetics and Genomics. 2006;276(2):101–12. 10.1007/s00438-006-0126-8. WOS:000240283200001. 16708235

[59] Pettersson ME, Johansson AM, Siegel PB, Carlborg O. Dynamics of adaptive alleles in divergently selected body weight lines of chickens. G3-Genes Genomes Genetics. 2013;3(12):2305–12. 10.1534/g3.113.008375. WOS:000328334500020. 24170737 PMC3852392

[60] Sarath G, Akin DE, Mitchell RB, Vogel KP. Cell-wall composition and accessibility to hydrolytic enzymes is differentially altered in divergently bred switchgrass (*Panicum virgatum* L.) genotypes. Applied Biochemistry and Biotechnology. 2008;150(1):1–14. 10.1007/s12010-008-8168-5. WOS:000256962600001. 18427744

[61] Zhong R, Yuan Y, Spiekerman JJ, Guley JT, Egbosiuba JC, Ye Z-H. Functional characterization of NAC and MYB transcription factors involved in regulation of biomass production in switchgrass (Panicum virgatum). Plos One. 2015;10(8). 10.1371/journal.pone.0134611. WOS:000359062300051. 26248336 PMC4527753

[62] Andersen JR, Zein I, Wenzel G, Darnhofer B, Eder J, Ouzunova M, et al Characterization of phenylpropanoid pathway genes within European maize (*Zea mays* L.) inbreds. Bmc Plant Biology. 2008;8 10.1186/1471-2229-8-2. WOS:000253964100001. 18173847 PMC2265712

[63] Xing Y, Frei U, Schejbel B, Asp T, Lubberstedt T. Nucleotide diversity and linkage disequilibrium in 11 expressed resistance candidate genes in *Lolium perenne*. Bmc Plant Biology. 2007;7 10.1186/1471-2229-7-43. WOS:000249620800001. 17683574 PMC1978496

[64] Zhu QH, Bennetzen JL, Smith SM. Isolation and diversity analysis of resistance gene homologues from switchgrass. G3-Genes Genomes Genetics. 2013;3(6):1031–42. 10.1534/g3.112.005447. WOS:000320768700011. 23589518 PMC3689800

[65] Zhang Y, Zalapa JE, Jakubowski AR, Price DL, Acharya A, Wei Y, et al Post-glacial evolution of *Panicum virgatum*: centers of diversity and gene pools revealed by SSR markers and cpDNA sequences. Genetica. 2011;139(7):933–48. 10.1007/s10709-011-9597-6. WOS:000293244900010. 21786028

[66] Vogel KP, Hopkins AA, Moore KJ, Johnson KD, Carlson IT. Winter survival in switchgrass populations bred for high IVDMD. Crop Science. 2002;42(6):1857–62. WOS:000181430200012.

[67] Costich DE, Friebe B, Sheehan MJ, Casler MD, Buckler ES. Genome-size variation in switchgrass (*Panicum virgatum*): flow cytometry and cytology reveal rampant aneuploidy. Plant Genome. 2010;3(3):130–41. 10.3835/plantgenome2010.04.0010. WOS:000208576300002.

[68] Aggarwal DD, Rashkovetsky E, Michalak P, Cohen I, Ronin Y, Zhou D, et al Experimental evolution of recombination and crossover interference in Drosophila caused by directional selection for stress-related traits. Bmc Biology. 2015;13 10.1186/s12915-015-0206-5. WOS:000365448100001. 26614097 PMC4661966

[69] Casler MD, Tobias CM, Kaeppler SM, Buell CR, Wang ZY, Cao PJ, et al The switchgrass genome: tools and strategies. Plant Genome. 2011;4(3):273–82. 10.3835/plantgenome2011.10.0026. WOS:000312661700011.

[70] Brummer EC, Casler MD. Improving selection in forage, turf, and biomass crops using molecular markers Molecular Breeding of Forage and Turf. 2009:193–209. 10.1007/978-0-387-79144-9_18. WOS:000261301000018.

[71] Liu H, Sorensen AC, Meuwissen THE, Berg P. Allele frequency changes due to hitch-hiking in genomic selection programs. Genetics Selection Evolution. 2014;46 10.1186/1297-9686-46-8. WOS:000333516800001. 24495634 PMC3942192

[72] Lorenzana RE, Lewis MF, Jung HJG, Bernardo R. Quantitative trait loci and trait correlations for maize stover cell wall composition and glucose release for cellulosic ethanol. Crop Science. 2010;50(2):541–55. 10.2135/cropsci2009.04.0182. WOS:000275564500012.

