# Supplementary figures and images for: Selection Signatures in Four Lignin Genes from Switchgrass Populations Divergently Selected for In Vitro Dry Matter Digestibility
Source: PLoS One. 2016 Nov 28;11(11):e0167005. doi: 10.1371/journal.pone.0167005 (PMC5125650; doi:10.1371/journal.pone.0167005)

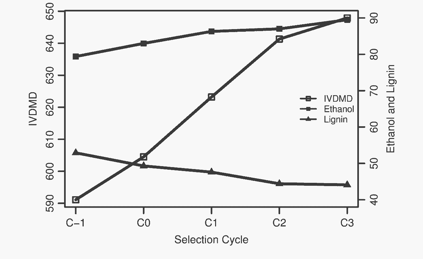

Supplement: S1 Fig — The figure is adapted from data of Vogel and others [12] for illustrative purpose only, not a replicate of published images. (TIF) [file pone.0167005.s001.tif]

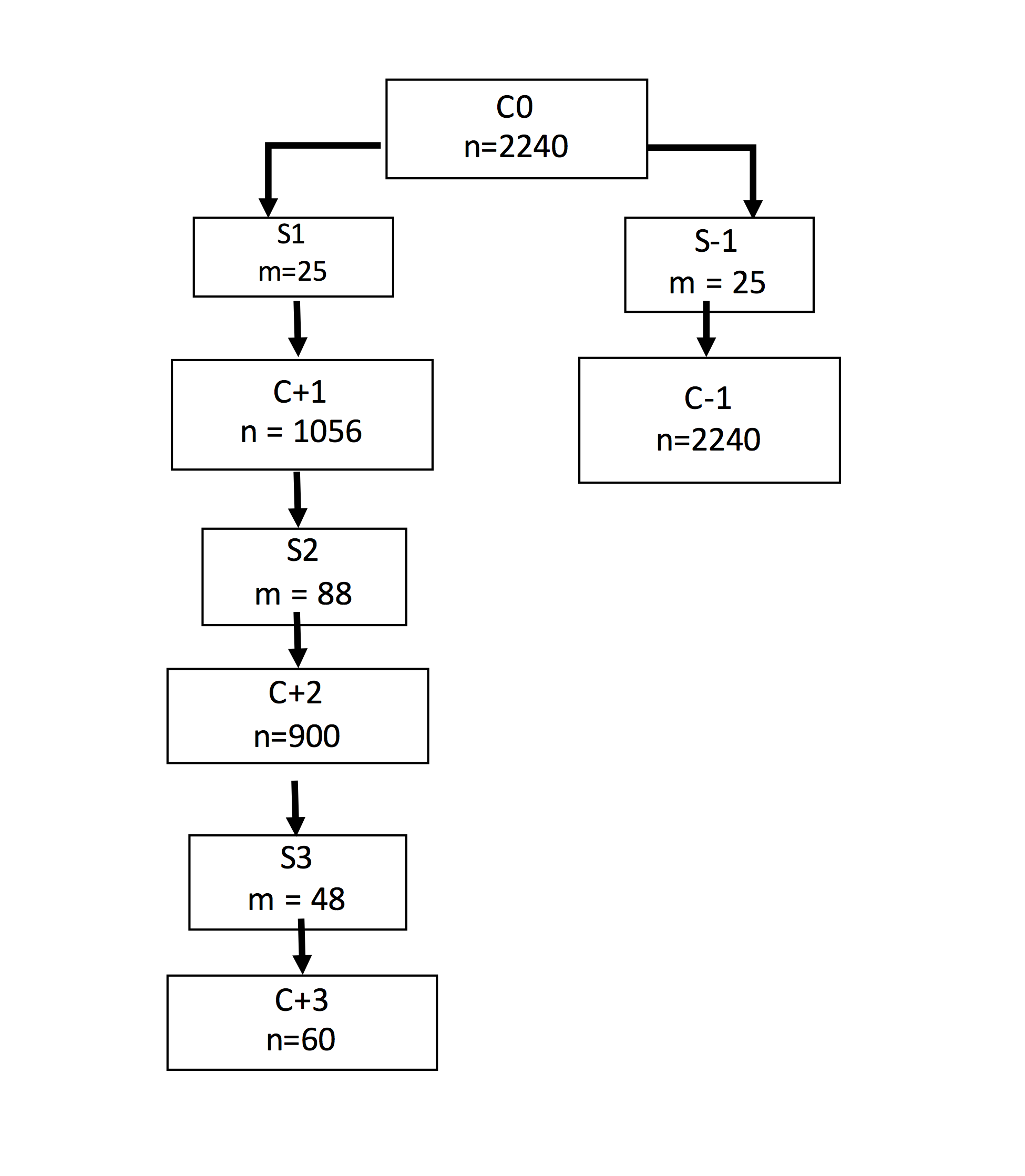

Supplement: S2 Fig — From the base population C0, one cycle of selection for low IVDMD and three cycles of selection for high IVDMD were conducted, resulting in four selected populations, C-1, C+1, C+2 and C+3. Population sizes are represented by n and the number of selected individuals by m for each group of selected individuals (S-1, S+1, S+2, and S+3). The figure is adapted from data of Vogel and others [12] for illustrative purpose only, not a replicate of published images. (TIFF) [file pone.0167005.s002.tiff]
