# Supplementary material for: Selection Signatures in Four Lignin Genes from Switchgrass Populations Divergently Selected for In Vitro Dry Matter Digestibility
Source: PLoS One. 2016 Nov 28;11(11):e0167005. doi: 10.1371/journal.pone.0167005 (PMC5125650; doi:10.1371/journal.pone.0167005)
Supplement: S1 Table — Switchgrass v3.1 genomic identifier were obtained from phytozome genome database by using our sequences as queries in BLAST. (DOCX) [file pone.0167005.s003.docx]

S1 Table. Summary information on allele sequences for four candidate genes obtained from the five divergent populations. Switchgrass v3.1 genomic identifier were obtained from phytozome genome database by using our sequences as queries in BLAST.

| Gene | NCBI accession | Sequence length (bp) | Primers for gene fragments  (5' to 3') | Switchgrass v3.1 |
| --- | --- | --- | --- | --- |
| COMT1 | FL749574; FL749575 | 1851 | F: GAGCTGGCCAACATGATCTC  R: TGACGAAGATGTCGTTCTCG | Pavir.1KG549300 |
| COMT2 | HQ645965 | 1593 | F: ACGCTCAAGAACGCCATC  R: TCCTCGAACTCCCTCTCGTA | Pavir.6KG070300 |
| CAD2 | GU045612 | 2782 | F: CCCCTACACCTACACCGTCA  R: CTCACCAGGTACGCGTCC | Pavir.1NG083600 |
| 4CL1 | EU491511 | 3845 | F: GAGATCAACAACAGCCAGCC  R: CCACAAGATTAGGTGCCCCT  Nested:  F: CACGCGTACTGCTTCGGC  R: CCCCTTCTGCTGCCATTTCA | Pavir.6KG154400 |
