# Supplementary material for: Selection Signatures in Four Lignin Genes from Switchgrass Populations Divergently Selected for In Vitro Dry Matter Digestibility
Source: PLoS One. 2016 Nov 28;11(11):e0167005. doi: 10.1371/journal.pone.0167005 (PMC5125650; doi:10.1371/journal.pone.0167005)
Supplement: S2 Table — (DOCX) [file pone.0167005.s004.docx]

S2 Table. The number of gene sequences sampled from each population allele pool.

| Gene | C-1 | C0 | C+1 | C+2 | C+3 | Total |
| --- | --- | --- | --- | --- | --- | --- |
| COMT1 | 49 | 188 | 60 | 46 | 53 | 396 |
| COMT2 | 44 | 193 | 51 | 44 | 51 | 383 |
| CAD2 | 56 | 212 | 63 | 68 | 57 | 456 |
| 4CL1 | 62 | 227 | 80 | 84 | 88 | 541 |
