# Supplementary material for: Selection Signatures in Four Lignin Genes from Switchgrass Populations Divergently Selected for In Vitro Dry Matter Digestibility
Source: PLoS One. 2016 Nov 28;11(11):e0167005. doi: 10.1371/journal.pone.0167005 (PMC5125650; doi:10.1371/journal.pone.0167005)
Supplement: S3 Table — (DOCX) [file pone.0167005.s005.docx]

S3 Table. Genetic diversity and haplotype diversity within the divergent populations for the four candidate genes.

| Gene | Population | π | sd(π) | π(nonsyn) | π(syn) | Pi(s) | H | Hd±SD | sd(Hd) |
| --- | --- | --- | --- | --- | --- | --- | --- | --- | --- |
| COMT1 | C-1 | 0.0040 | 0.0029 | 0.0000 | 0.0039 | 0.0067 | 14 | 0.796 | 0.054 |
| COMT1 | C0 | 0.0033 | 0.0004 | 0.0000 | 0.0036 | 0.0052 | 14 | 0.662 | 0.034 |
| COMT1 | C+1 | 0.0040 | 0.0042 | 0.0000 | 0.0037 | 0.0062 | 17 | 0.705 | 0.065 |
| COMT1 | C+2 | 0.0059 | 0.0027 | 0.0000 | 0.0040 | 0.0091 | 20 | 0.853 | 0.003 |
| COMT1 | C+3 | 0.0063 | 0.0005 | 0.0000 | 0.0039 | 0.0067 | 14 | 0.852 | 0.001 |
| COMT2 | C-1 | 0.0058 | 0.0003 | 0.0011 | 0.0068 | 0.0096 | 10 | 0.879 | 0.029 |
| COMT2 | C0 | 0.0060 | 0.0070 | 0.0011 | 0.0061 | 0.0096 | 38 | 0.930 | 0.007 |
| COMT2 | C+1 | 0.0054 | 0.0003 | 0.0010 | 0.0076 | 0.0088 | 15 | 0.857 | 0.034 |
| COMT2 | C+2 | 0.0057 | 0.0003 | 0.0010 | 0.0055 | 0.0092 | 7 | 0.777 | 0.045 |
| COMT2 | C+3 | 0.0058 | 0.0002 | 0.0011 | 0.0068 | 0.0096 | 11 | 0.838 | 0.038 |
| 4CL1 | C-1 | 0.0028 | 0.0002 | 0.0015 | 0.0021 | 0.0031 | 21 | 0.903 | 0.024 |
| 4CL1 | C0 | 0.0022 | 0.0001 | 0.0015 | 0.0014 | 0.0024 | 53 | 0.809 | 0.024 |
| 4CL1 | C+1 | 0.0028 | 0.0002 | 0.0018 | 0.0025 | 0.0031 | 15 | 0.733 | 0.039 |
| 4CL1 | C+2 | 0.0020 | 0.0003 | 0.0010 | 0.0018 | 0.0022 | 13 | 0.528 | 0.067 |
| 4CL1 | C+3 | 0.0035 | 0.0001 | 0.0013 | 0.0028 | 0.0041 | 30 | 0.872 | 0.028 |
| CAD2 | C-1 | 0.0043 | 0.0002 | 0.0013 | 0.0112 | 0.0049 | 22 | 0.918 | 0.025 |
| CAD3 | C0 | 0.0041 | 0.0001 | 0.0013 | 0.0105 | 0.0047 | 53 | 0.913 | 0.012 |
| CAD4 | C+1 | 0.0041 | 0.0003 | 0.0014 | 0.0109 | 0.0049 | 20 | 0.850 | 0.047 |
| CAD5 | C+2 | 0.0038 | 0.0003 | 0.0011 | 0.0091 | 0.0044 | 14 | 0.885 | 0.016 |
| CAD6 | C+3 | 0.0041 | 0.0001 | 0.0015 | 0.0113 | 0.0046 | 25 | 0.921 | 0.026 |
